# Early Non-Response to Neoadjuvant Chemotherapy Will Increase the Recurrence of Cervical Cancer: A Systematic Review

**DOI:** 10.3390/biomedicines13082016

**Published:** 2025-08-19

**Authors:** Shiqing Huang, Runfeng Yang, Li Yang, Shiyi Kong, Kecheng Huang

**Affiliations:** 1 Department of Obstetrics and Gynecology, Tongji Hospital, Tongji Medical College, Huazhong University of Science and Technology, Wuhan 430030, China; 2 National Clinical Research Center for Obstetrics and Gynecology, Cancer Biology Research Center (Key Laboratory of the Ministry of Education), Tongji Hospital, Tongji Medical College, Huazhong University of Science and Technology, Wuhan 430030, China; 3 Department of Gynecologic Oncology, Hubei Cancer Hospital, Tongji Medical College, Huazhong University of Science and Technology, Wuhan 430079, China

**Keywords:** cervical cancer, neoadjuvant chemotherapy (NACT), early non-response, long-term survival, systematic review

## Abstract

**Objectives**: Cervical cancer remains a significant global health burden for women. While neoadjuvant chemotherapy (NACT) has emerged as a potential treatment option, the prognostic implications of early non-response to NACT remain inadequately characterized. This systematic review aims to elucidate the association between early non-response to NACT and long-term disease-free survival (DFS) in cervical cancer patients. **Methods**: A comprehensive systematic review was conducted following PRISMA guidelines. PubMed, Embase, Elsevier, Springer, EBSCO, and Cochrane Library were systematically searched to identify eligible studies. Pooled hazard ratios (HRs) for DFS with 95% confidence intervals (CIs) were calculated using R software (version 4.5.1). Heterogeneity was assessed via Cochran’s Q test and I^2^ statistics. Publication bias was evaluated using funnel plots, Begg’s test, Egger’s test, and trim-and-fill methods. Sensitivity analyses further validated result robustness. **Results**: Eleven studies (n = 2064 patients; 1546 responders, 518 non-responders) met inclusion criteria. The pooled early non-response rate ranged from 13% to 39%. Early non-response significantly correlated with poorer DFS (HR = 3.29, 95% CI 2.35–4.62). Subgroup analyses by response criteria showed HRs of 2.94 (95% CI 1.72–5.03) for WHO criteria and 4.00 (95% CI 2.52–6.34) for RECIST criteria. No significant publication bias was detected (Begg’s *p* = 0.35; Egger’s *p* = 0.28). Sensitivity analyses and trim-and-fill adjustments confirmed result stability. **Conclusions**: Early non-response to NACT predicts worse DFS in women with cervical cancer. These findings proposed the need for large-scale or prospective studies to validate the prognostic value of early non-response and optimize treatment strategies for non-responders. Future prospective trials with standardized protocols are essential to validate these findings and establish criteria for optimizing patient selection for NACT-based therapeutic strategies.

## 1. Introduction

Cervical cancer is one of the most common malignant tumor diseases for women [1]. For cervical cancer patients presenting with bigger tumors (measuring between 2 and 4 cm), a viable alternative treatment pathway involves administering neoadjuvant chemotherapy prior to undergoing fertility-preserving surgery [2,3]. Furthermore, studies indicate that NACT not only reduces tumor volume and metastatic potential but also enhances surgical feasibility [4,5]. Clinical evidence suggests that patients responsive to NACT who undergo subsequent surgery exhibit improved survival outcomes compared to non-responders.

Not limited to cervical cancer, scientists are also exploring fertility-preserving treatment approaches for other gynecological malignancies [6,7] while simultaneously pursuing medical strategies to reduce recurrence rates [8,9]. Current clinical management of cervical cancer follows the FIGO staging guidelines, and chemo-radiotherapy (CCRT) is established as a standard treatment. Neoadjuvant chemotherapy (NACT) has emerged as an alternative strategy over recent decades [4,5]. NACT demonstrates potential benefits in tumor volume reduction, enhancing surgical feasibility, and enabling fertility preservation in younger patients [2]. Some studies revealed that preoperative NACT reduced adjuvant radiation requirements in cervical cancer patients undergoing radical surgery. Although NACT response has been established as a reliable prognostic marker in malignancies such as locally advanced breast cancer (LABC), its predictive value in locally advanced cervical cancer (LACC) remains insufficiently characterized. Not all early-stage gynecological cancers benefit meaningfully from neoadjuvant chemotherapy, and its use remains debated for certain cases [10]. Our systematic evaluation of clinical studies demonstrates variable response rates to NACT in LACC, collectively suggesting tumor chemo-sensitivity. Notably, multiple studies identified significant correlations between positive clinical response to NACT and improved overall survival (OS) as well as disease-free survival (DFS). These findings position NACT responsiveness as a potential dual-purpose biomarker, capable of both prognosticating survival outcomes and guiding personalized adjuvant therapy decisions [4].

However, prognostic predictions for NACT non-responders remain inconsistent across existing studies. Although previous articles showed statistical homogeneity and rigorous methodology, limitations warrant consideration. Most studies were retrospective in design, with many of them originating from Asia, probably introducing potential selection bias. Significant heterogeneity in chemotherapy regimens may exist, predominantly platinum-based protocols supplemented by agents such as irinotecan, docetaxel, nedaplatin, bleomycin, vincristine, and 5-FU. Such pharmacological diversity may contribute to outcome variability. This review aims to synthesize updated data on cervical cancer patients treated with NACT to evaluate the impact of early non-response on clinical prognosis. This synthesis is designed to confirm that NACT responsiveness predicts favorable prognosis in LACC.

## 2. Materials and Methods

Literature Review. A comprehensive literature search was conducted up to December 2024 using the online databases PubMed, Embase, Elsevier, Springer, EBSCO, and Cochrane Library. Boolean operators (“AND”, “OR”, and “NOT”) were applied to intersect these domains, generating a structured search query. Secondary searches supplemented initial results by reviewing bibliographies of relevant publications and prior systematic reviews or clinical trials addressing similar therapeutic protocols. No temporal restrictions were imposed on publication dates to ensure comprehensive coverage of historical and contemporary evidence. This systematic review adhered to the Preferred Reporting Items for Systematic Reviews and Meta-Analyses (PRISMA) and Meta-analysis Of Observational Studies in Epidemiology (MOOSE) guidelines [11,12]. Search terms included “cervical cancer”, “cervical carcinoma”, “cervical neoplasia”, “neoadjuvant chemotherapy or NACT or preoperative chemotherapy”, and “prognosis or survival” restricted to the titles, abstracts, or keywords of articles. Medical Subject Headings (MeSH) terms were also utilized to refine the search. Additionally, references of eligible publications were manually screened to identify further relevant studies.

Eligible Criteria. Eligible studies in this analysis also need to meet all of the following criteria: enrolled women with cervical cancer who underwent NACT; availability of stratified survival outcomes (clinical responders vs. non-responders); reported detailed survival outcomes for short-term responders; provided sufficient data to calculate hazard ratios (HRs) and 95% confidence intervals (CIs), were published in English as full-text articles; Eligible studies were further scrutinized to evaluate stage-specific outcomes, including tumor response rates, surgical resectability improvements, and survival metrics in cervical cancer patients [13,14].

Exclusion Criteria. If the endpoint evaluated was the achievement of optimal pathologic response following neoadjuvant chemotherapy, the studies were excluded. Pathological responses to neoadjuvant chemotherapy were categorized according to standardized criteria: complete response (CR) denoted total eradication of cervical tumor with absence of nodal involvement; optimal partial response (OPR) was characterized by residual disease demonstrating stromal invasion ≤3 mm; and suboptimal partial response (SOPR) was defined as persistent cervical lesions with stromal infiltration >3 mm. Disease stabilization (SD) referred to static tumor dimensions without volumetric or invasive progression, while progressive disease (PD) indicated measurable tumor expansion or metastatic dissemination.

Qualitative assessment and data extraction. Two independent researchers evaluated the methodological quality of included studies using the Newcastle–Ottawa Scale (NOS) [15]. Two additional authors extracted data using standardized protocols, with discrepancies resolved through discussion with a senior investigator. Extracted data included the following: first author, publication year, country, sample size, follow-up duration, FIGO stage, clinical response rates (complete/partial remission), NACT cycles, and survival outcomes. To address heterogeneity, clinical responses were categorized using either the RECIST (Response Evaluation Criteria in Solid Tumors) or WHO criteria [16].

Statistical Analysis. The HR and 95% CI were used to assess the relationship between patients’ short-term responses to NACT and long-term DFS rates [13,14]. Heterogeneity was evaluated using Cochran’s Q-test (significance threshold: *p* < 0.10) and the I^2^ statistic (low/moderate heterogeneity: I^2^ < 50%). Fixed-effects models (Mantel–Haenszel) were applied for I^2^ < 50%, while random-effects models (DerSimonian–Laird) were used for I^2^ ≥ 50% [17]. A fixed-effect model was applied for low-heterogeneity studies, while a random-effects model was used for high-heterogeneity cases [18]. When significant heterogeneity exists, random-effects model will be used based on the DerSimonian–Laird method [19]. Sensitivity analyses tested the robustness of HR estimates [20]. Publication bias was assessed via funnel plots, trim-and-fill analysis, and statistical tests (Begg’s and Egger’s methods) [21,22]. Institutional review board approval was formally waived under applicable regulations, as this investigation exclusively involved secondary analysis of aggregated data from peer-reviewed publications. The complete research dataset will be accessible to qualified investigators through the journal’s editorial office upon formal request, specifically to permit independent validation of findings or facilitate supplementary analytical verification in accordance with open science principles. The R Language (version 4.5.1) was applied for statistical analysis.

## 3. Results

### 3.1. Features of Qualified Studies Related to HRs

**Figure 1:**
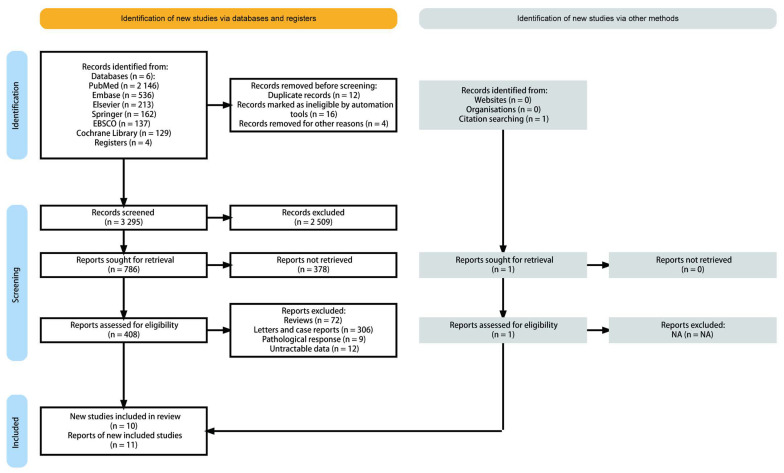
PRISMA flow diagram and exclusion criteria.

We identified related publications from the bibliography of the PubMed, Embase, Elsevier, Springer, EBSCO, and Cochrane Library, as well as related articles. After reviewing the title and summary, the full text of the remaining 408 potential articles was assessed. We also excluded 398 articles, as they did not meet the selection criteria (Figure 1). Eventually 11 studies derived from 10 publications were enrolled in the final pooling analysis.

Table 1 showed all the basic key features that were collected in the present study. There was a total number of 2064 cases from 11 studies that provided clinical response data from NACT [23,24,25,26,27,28,29,30,31,32]. Most of the articles reported patients with FIGO IB to IIB cervical cancer.

### 3.2. Combination Result for Included Studies

**Figure 2:**
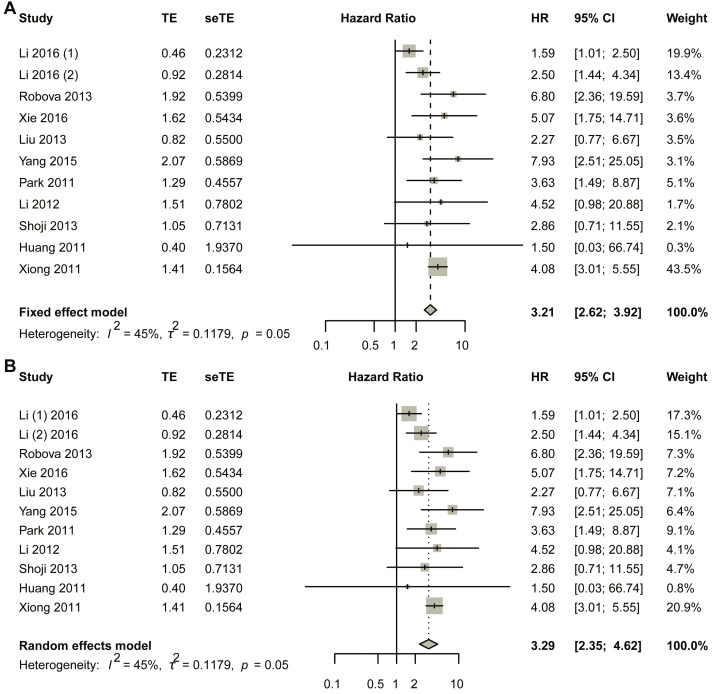
Title: Comparative Analysis of Hazard Ratios for Disease-Free Survival (DFS) Between Non-Responders and Responders. Legend: The forest plot presents aggregated hazard ratios (HRs) derived from a pooled analysis comparing DFS outcomes between non-responder and responder subgroups in cervical cancer patients receiving neoadjuvant chemotherapy. Two distinct statistical models were employed: fixed-effect model (**A**) assumes homogeneity of treatment effects across studies; random-effects model (**B**) accounts for potential inter-study variability. A total of 11 studies in 10 articles were pooled [23,24,25,26,27,28,29,30,31,32].

The fixed-effect model was first used (Figure 2A). The comprehensive analysis showed that the short-term response to NACT led to a better survival result: early non-response had a negative relationship with DFS, and non-response was more likely to reduce the DFS rate (HR, 3.2054; 95% CI, 2.6185–3.9239). Heterogeneous tests showed that I^2^ equaled to 45.2% (95% CI, 0.0–72.8%), tau equaled to 0.3434 (95% CI, 0.0000–0.7569), tau-square equaled to 0.1179 (95% CI, 0.0000–0.5729), and H-value equaled to 1.35 (95% CI, 1.00–1.92); meanwhile, Cochrane Q test showed that *p* equaled to 0.0348 with Q-value of 20.86 at the degree of freedom of 10 (Figure 2A). To assess potential publication bias, a funnel plot was generated for visual inspection (Figure S1A). Since no significant asymmetry was detected, Egger’s test was conducted to quantitatively evaluate funnel plot asymmetry (Figure S1B). The results indicated a *p*-value of 0.7216 (*t*-value = 0.3676), with an intercept of 1.0831 and a bias estimate of 0.2857 (standard error = 0.7772). Additionally, Begg’s test yielded a *p*-value of 0.9379 (*z*-value = 0.07785), further supporting the absence of substantial bias (Figure S1C). To further validate these findings, Duval and Tweedie’s trim-and-fill method was applied, identifying one missing study. After adjustment, the random-effects model produced a hazard ratio (HR) of 3.1142 (95% CI: 2.5518–3.8005; *p* < 0.0001; *z* = 11.18). Notably, the pooled estimate remained robust, with the lower 95% confidence limit unchanged at 2.5518 (Figure S1D). Sensitivity analysis was also performed using a fixed-effects model (Figure S2).

In the random effects model, the pooled HR was 3.2932 (95% CI, 2.3488–4.6173; *p* < 0.0001) with *z*-value of 6.91 (Figure 2B). Funnel plot was made to visually observe the publication bias that may exist (Figure S3A). As no obvious bias had been observed from the funnel plot (Figure S3B), Egger’s test was employed to test the asymmetry of funnel plot; the test showed that *p*-value equaled to 0.7216 with t-value of 0.3676; sample estimates showed that intercept equaled to 1.0831, and bias equaled to 0.2857 with standard error of 0.7772. Begg’s test was also made, and the result showed *p*-value equaled to 0.9379 with z-value of 0.07785 (Figure S3C). Duval and Tweedie’s trim-and-fill method was also employed to detect publication bias, and the result showed that with one added study, the random-effects model yielded a new result of HR = 3.1114 (95% CI 2.2181–4.3644; *p* < 0.0001; *z* = 6.57). Still, the combined result did not change much, with the lower limit of 95% CI of 2.2181 2181 (Figure S3D). At the same time, the random-effects model was also used in the sensitivity analysis, and the result showed that the largest value of HR was 3.82, while the smallest was 3.09. The lower limit of confidence intervals was 2.13 and the upper limit confidence intervals was 5.16; all the pooled HRs were statistically significant (Figure S4).

### 3.3. Sub-Group Analysis Based on Response Criterion

**Figure 3:**
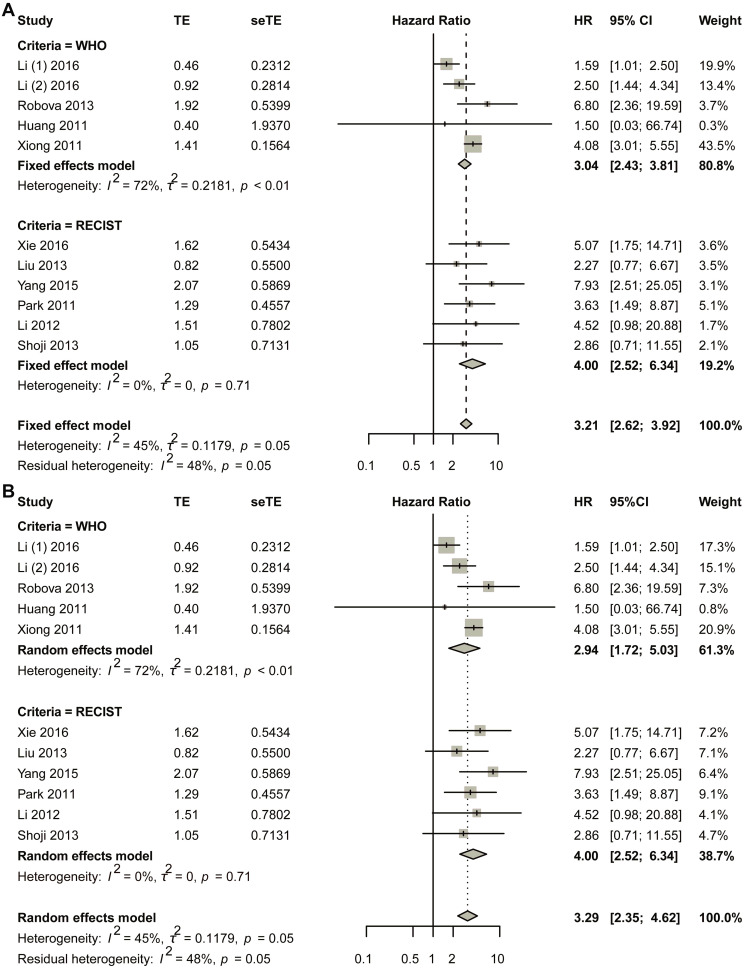
Title: Comparative analysis of HR between non-responders and responders across subgroups stratified by varying response criteria. Legend: The forest plot presents pooled HRs. Two methodological approaches were employed: (**A**) fixed-effect model; (**B**) random-effects model. A total of 11 studies in 10 articles were pooled [23,24,25,26,27,28,29,30,31,32].

The initial pooled analysis employed a fixed-effects model (Figure 3A), revealing a hazard ratio (HR) of 3.04 (95% confidence interval [CI]: 2.43–3.81) for the WHO group. Visual assessment of the funnel plot indicated no significant asymmetry (Figure S5A). Statistical evaluation used Egger’s test (Figure S5B), as well as Begg’s test (Figure S5C), collectively suggesting minimal publication bias. Duval and Tweedie’s trim-and-fill analysis revealed no obvious bias (k = 5; 0 studies imputed; Figure S5D). Sensitivity analysis confirmed result stability (Figure S6).

Considering the heterogeneity existing in the WHO group, the random-effects model was also used in the pooled analysis (Figure 3B). The HR for the WHO group was 2.94 (95% CI 1.72–5.03; *p* < 0.0001). The funnel plot showed that no obvious asymmetry was observed (Figure S7A). Begg’s test showed that the intercept was 1.19, with *p* = 0.86 (Figure S7B); Egger’s test showed that *p*-value was 1 (Figure S7C); Duval and Tweedie’s trim-and-fill method plot showed k = 5 with zero added studies (Figure S7D). Sensitivity analysis was conducted, and the plot showed that the lower limit of confidence intervals was 1.38 (Figure S8).

The fixed-effects model demonstrated a statistically significant hazard ratio (HR) of 4.00 (95% confidence interval [CI]: 2.52–6.34) for the RECIST criteria-defined population (Figure 3B). Comprehensive publication bias assessment was conducted through multiple complementary methodologies. Visual evaluation of the funnel plot symmetry (Figure S9A) revealed no substantial asymmetry, suggesting balanced representation of studies across effect sizes. This finding was quantitatively confirmed through formal statistical testing: Egger’s regression test (Figure S9B) and Begg’s rank correlation method (Figure S9C) both yielded non-significant results (*p* > 0.05), indicating absence of small-study effects. The robustness of these conclusions was further verified using Duval and Tweedie’s trim-and-fill algorithm, which estimated zero missing studies (Figure S9D), reinforcing the completeness of the included literature. Methodological rigor was enhanced through sensitivity analyses examining the stability of effect estimates under varying analytical conditions (Figure S10). The lower boundary of the 95% confidence intervals consistently remained above 1.00 across all sensitivity scenarios, demonstrating that the observed association was not contingent upon specific modeling assumptions. This multi-faceted analytical approach combining visual inspection, statistical testing, and sensitivity evaluation provides strong evidence for the reliability of the pooled estimate. The concordance among these diverse assessment methods substantiates the validity of the reported HR for the RECIST group.

In the random-effects model, the HR for the RECIST group was 4.00 (95% CI 2.52–6.34) (Figure 3B). The funnel plot showed that no obvious asymmetry was observed (Figure S11A). Egger’s test showed that *p* was 1 (Figure S11B); Begg’s test showed that *p* was 0.8568 (Figure S11C); the trim-and-fill method showed that no additional study was added (Figure S11D). Sensitivity analysis showed that the lower limit of confidence intervals was larger than 1.00 (Figure S12).

### 3.4. Sub-Group Analysis Based on Different Areas

**Figure 4:**
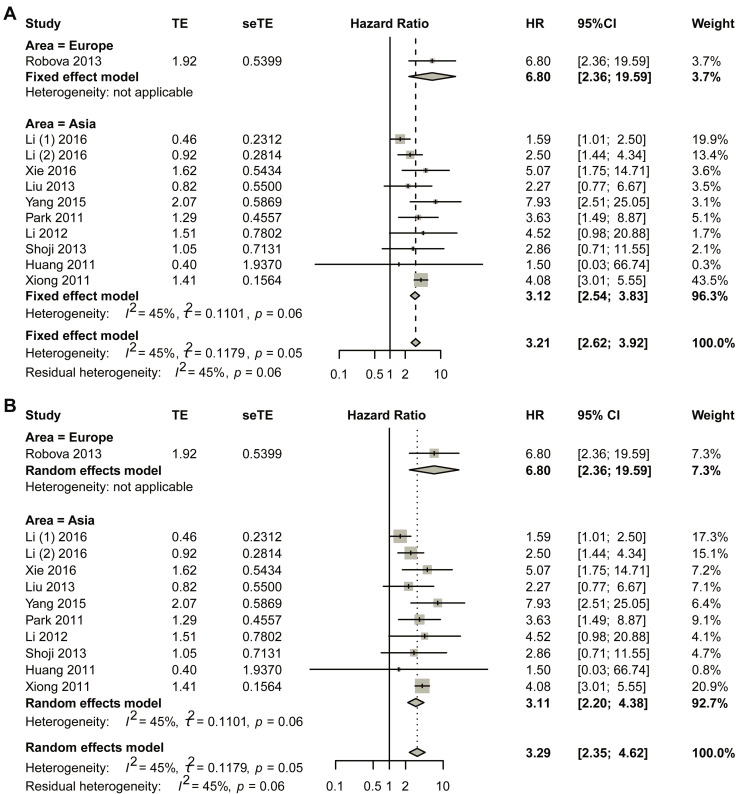
Title: Comparative assessment of hazard ratios (HRs) between non-responders and responders across geographic subgroups. Legend: (**A**) fixed-effect pooled analysis; (**B**) random-effects pooled analysis. Individual study estimates (data points) represent HR comparisons of non-responders versus responders in neoadjuvant chemotherapy (NACT)-treated cervical cancer cohorts. Data point dimensions reflect study-specific weights. Diamond markers indicate pooled HR estimates with corresponding 95% CI. A total of 11 studies in 10 articles were pooled [23,24,25,26,27,28,29,30,31,32].

Sub-group analysis was performed based on different areas by first using the fixed-effect model (Figure 4). For studies in European countries, one study was included in the final analysis, and the HR was 6.80 (95% CI, 2.36–19.59). Asian populations revealed a pooled hazard ratio (HR) of 3.12 (95% CI, 2.54–3.83). To ensure the robustness of these results, comprehensive bias assessment was conducted through multiple complementary approaches. Visual inspection of funnel plots (Figure S13A) demonstrated satisfactory symmetry across effect size estimates, suggesting minimal publication bias in the included studies. This graphical assessment was further validated through quantitative analyses, including Egger’s regression test (Figure S13B) and Begg’s rank correlation method (Figure S13C), both yielding non-significant *p*-values (*p* > 0.05). The consistency between these different assessment approaches strengthens confidence in the validity of the pooled estimate. Publication bias was assessed through the trim-and-fill method, which revealed no significant asymmetry (Figure S13D). These findings were further supported by sensitivity analyses presented in Figure S14. Sensitivity analyses (Figure S14) were systematically performed to evaluate the stability of the results under varying analytical conditions. These included sequential exclusion of individual studies and alternative model specifications. The HR estimates remained consistently across all sensitivity scenarios. This methodological rigor confirms that the observed association is not unduly influenced by any single study or analytical approach. The comprehensive bias assessment framework employed in this analysis, combining both visual and statistical methods, provides strong evidence for the reliability of the reported effect size in Asian populations.

The HR for Asian studies was 3.11 with 95% CI of 2.20–4.38 using the random effect model. For the Asian group, visual symmetry of the funnel plots was performed to estimate publication bias, and no obvious publication bias was observed (Figure S15). Sensitivity analysis is shown in Figure S16.

### 3.5. Sub-Group Analysis for Asian Studies

**Figure 5:**
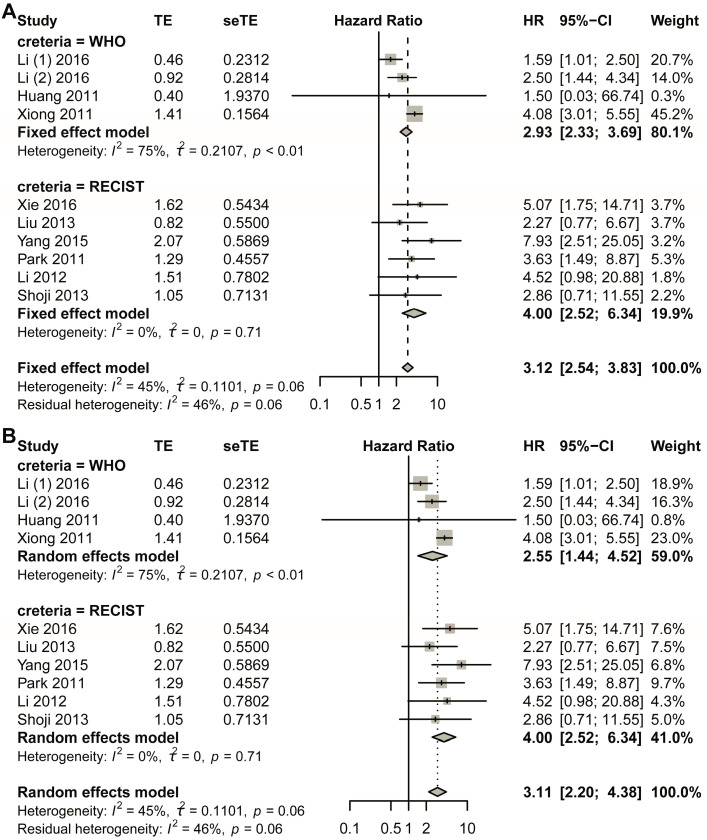
Title: HR disparities between treatment non-responders and responders in Asian population. Legend: This analysis evaluates pooled HRs stratified by response assessment criteria in Asian cervical cancer patients receiving neoadjuvant chemotherapy (NACT). Two distinct methodological approaches were implemented: (**A**) fixed-effect model; (**B**) random-effects model. A total of 10 studies in nine articles were pooled [23,24,25,26,27,28,29,30,31,32].

The analysis for the RECIST subgroup has been described in Section 3.3. The fixed-effect model was first used (Figure 5) in the WHO sub-group. The forest plot showed that non-response was a risk factor for DFS (HR, 2.93; 95% CI, 2.33–3.69). The heterogeneity test showed that value of I^2^ was 74.9%, and *p* for Cochran’s Q was 0.0076. Publication bias was assessed through visual inspection of funnel plot symmetry, which revealed no apparent bias (Figure S17A). Further evaluation using Egger’s and Begg’s tests confirmed the absence of significant publication bias, with *p*-values of 0.58 and 1.00, respectively (Figure S17B,C). The trim-and-fill method by Duval and Tweedie identified two missing studies that were subsequently included in the pooled analysis (Figure S17D), yielding an adjusted HR of 3.64 (95% CI: 2.96–4.46). Sensitivity analysis demonstrated robust findings, with the lower confidence interval boundary remaining at 1.34 (Figure S18).

The random-effects model was also performed in the analysis. The HR for the WHO sub-group was 2.55 with 95% CI of 1.44–4.52 (Figure 5). The visual symmetry of the funnel plots was evaluated to estimate publication bias, and no obvious publication bias was observed (Figure S19). Meanwhile, Egger’s test and Begg’s test were performed to detect publication bias, and the symmetrical funnel chart showed that there was no significant publication bias in the study, with *p* = 0.58 for Egger’s test and *p* = 1 for Begg’s test (Figure S19B,C). Duval and Tweedie’s trim-and-fill plot was also generated, and two additional studies were added to the present combined analysis (Figure S19D); still, the new HR was 3.445 (95% CI, 1.86–6.39). Sensitivity analysis was conducted, and the final results showed that the lower limit of confidence intervals was 1.07 (Figure S20).

## 4. Discussion

This review demonstrates that early non-response to neoadjuvant chemotherapy (NACT) in cervical cancer patients is significantly associated with poorer long-term prognosis, particularly reduced disease-free survival (DFS) and increased recurrence risk. These findings align with the existing literature and validate the predictive value of early treatment response. Notably, both the WHO and RECIST criteria yielded consistent results, promoting the robustness of non-response as a prognostic indicator.

Cervical carcinoma, a malignancy originating in the cervical epithelium, is predominantly associated with persistent human papillomavirus (HPV) infection. As one of the most prevalent gynecologic malignancies globally, it remains one of the leading contributors to morbidity and mortality among women, particularly in low- to middle-income nations [33,34]. Current therapeutic approaches for cervical cancer continue to evolve, with ongoing debates regarding optimal management strategies for specific tumor stages, including locally advanced cervical cancer (LACC). Over the past two decades, its global incidence has demonstrated a concerning upward trajectory. Tumors exceeding 4 cm in diameter, classified as bulky cervical carcinomas (such as stages IB3, IIA2, and IIB), present unique clinical challenges due to therapeutic controversies and poorer prognostic outcomes compared to earlier-stage disease. These advanced lesions exhibit higher rates of local recurrence and lymphatic metastasis relative to IB2 or lower-stage tumors.

Current clinical guidelines lack consensus regarding optimal management of stage IB3-IIB disease. Therapeutic options under consideration include primary chemoradiation, radical hysterectomy, or neoadjuvant chemotherapy followed by surgical intervention. The National Comprehensive Cancer Network (NCCN) outlines three potential strategies: immediate radical hysterectomy, neoadjuvant chemotherapy preceding surgery, or concurrent chemoradiotherapy. Common chemotherapeutic regimens incorporate agents such as cisplatin, paclitaxel, topotecan, vinorelbine, gemcitabine, and ifosfamide. Emerging evidence suggests potential benefits of neoadjuvant approaches. Previous studies demonstrated improved prognoses in LACC patients receiving weekly paclitaxel/carboplatin regimens followed by radical surgery. Neoadjuvant chemotherapy may enhance surgical resectability by reducing tumor bulk, minimizing parametrial infiltration, and decreasing lymphatic metastasis risk, thereby potentially improving survival rates, quality of life, and reducing postoperative radiation requirements [4].

We have validated previous findings made by other researchers. Park and colleagues also reported results from a phase II clinical trial, and they found that responders to NACT had a higher 5-year DFS compared with non-responders; they also conducted a multiple variable analysis using the Cox proportional hazards regression model and found that the HR was 3.63 with 95% CI of 1.49–8.89 when non-responders were compared with responders [27]. Hu and colleagues similarly found that the non-responder group had lower DFS and overall survival rates compared with the responder group and the radical surgical group [35]. Li and colleagues conducted a retrospective study, and they found that responding to NACT ahead of operation might be regarded as an independent protective factor among cervical cancer patients at early FIGO stage with bulky tumor diseases. They also found that, compared with non-responders, responders of NACT had lower lymph-vascular space invasion rates, less deep stromal invasion, lower lymph node metastasis rates, and reduced need for adjuvant radiotherapy. Meanwhile, they achieved improved 5-year progression-free survival and 5-year overall survival rates; they also performed multiple variable Cox proportional regression hazards regression analyses suggesting that responsiveness to NACT might be regarded as an independent protective factor for both progression-free and overall survival [24]. For clinical studies failing to document time-to-event analyses through either graphical Kaplan-Meier estimators or Cox proportional hazards models, an institutional data algorithm system was used. 

Homogeneity in response and non-response rates across studies may stem from two factors. First, the majority of NACT regimens for locally advanced cervical cancer (LACC) are platinum-based (such as carboplatin, cisplatin, nedaplatin, and so on), which are standardized and widely adopted, minimizing variability in therapeutic effects. Second, the RECIST criteria, a precise tool for evaluating tumor response, likely reduced heterogeneity by ensuring uniform outcome assessments. This systematic literature review was conducted on December 2024, to identify original research articles evaluating the efficacy of neoadjuvant chemotherapy, specifically the platinum-based regimens, in managing bulky cervical carcinomas classified as LACC. Previous studies lacked direct relevance to the specified tumor stages or failed to address the pharmacodynamic interplay between chemotherapy and cervical cancer pathophysiology. Recently, more and more particular emphasis was placed on analyzing pharmacokinetic properties and pharmacodynamics mechanisms underlying this regimen’s effects on angiogenesis suppression, mitotic inhibition, and tumor microenvironment destabilization. Neoadjuvant chemotherapy (NACT) involves preoperative systemic treatment aimed at tumor volume reduction prior to definitive surgical intervention. This approach may improve life expectancy compared to adjuvant chemotherapy by mitigating tumor genetic heterogeneity. Clinical studies report that NACT followed by surgery enhances progression-free survival (PFS) and disease-free survival (DFS). Additionally, NACT facilitates surgical planning optimization, enables real-time monitoring of therapeutic response, and allows regimen adjustments during treatment initiation or upon suboptimal tumor response. Historically, cisplatin, paclitaxel, and ifosfamide have demonstrated activity against cervical carcinomas, with cisplatin achieving a 20% response rate. Combination regimens targeting heterogeneous tumor cell populations have shown enhanced therapeutic efficacy. Multi-agent protocols employing mechanistically distinct drugs reduce chemoresistance risks while minimizing individual agents’ toxicity [36].

Recent investigations highlight the carboplatin–paclitaxel regimen as a viable alternative to cisplatin–paclitaxel in recurrent disease, demonstrating comparable efficacy with improved tolerability. Carboplatin’s toxicity profile demonstrates reduced incidence of nausea, neurotoxicity, and nephrotoxicity relative to cisplatin. Preoperative neoadjuvant chemotherapy (NACT) serves as a cytoreductive strategy prior to definitive surgical intervention or radiotherapy. In locally advanced cervical carcinomas, administration of 2–3 NACT cycles has demonstrated enhanced resectability rates through tumor volume reduction and suppression of micrometastatic spread. Clinical evidence indicates that NACT-mediated tumor downsizing facilitates complete macroscopic resection while potentially mitigating lymphatic metastasis and parametrial invasion. Some investigators have further validated the therapeutic utility of NACT, revealing favorable response rates with manageable hematological or non-hematological toxicities.

Robova and colleagues conducted a prospective study from 1998 to 2009, which enrolled 154 patients; and they found that the mortality rate of responders was significantly decreased compared with non-responders [28]. Xie and colleagues conducted research among cervical cancer patients with NACT prior to surgery; and they found that responders to NACT had lower 5-year disease-free survival, with *p* = 0.0003 for the univariate Cox proportional regression model and HR = 5.072 for multivariate Cox proportional regression model. Meanwhile, they found the non-responders also had significantly lower 5-year overall survival (*p* = 0.022) for the univariate Cox proportional regression model. The multivariate Cox proportional regression model also showed that the overall survival was not in favor of non-responders (HR, 2.225; 95%CI, 0.813–6.092) [30]. Yang and colleagues conducted research on cervical cancer patients who underwent NACT consisted of paclitaxel plus carboplatin. And they found that cervical cancer patients could benefit from NACT as long as they responded to the NACT regimen. They found the early non-responders showed significantly lower overall survival rates and progression free survival rates when compared with early responders; the group of responders even showed survival advantages compared with the group of primary radical surgery. After using log-rank test, they observed that the overall survival of early responders was statistically longer than early non-responders, and they also observed similar result that early responders achieving statistically longer progression survival than early non-responders [32]. Xiong and colleagues conducted a retrospective study and found that early response could be regarded as an indicator to long-term prognosis; they observed that early response to NACT was the only significant prognostic factor of survival under the condition of multiple variable Cox proportional hazards regression analysis [31]. From the pooled results, we note that the non-response and response rates induced by the chemotherapy regimens are very similar. We think there are several reasons. Firstly, regarding regimens for LACC, most NACT regimens are platinum-based and widely used drugs. Secondly, chemotherapy drugs such as carboplatin, nedaplatin, and cisplatin are all platinum anticancer drugs, which is also reflected by the quite mild differences between the studies included. Thirdly, the RECIST criteria are a much more accurate tool, resulting in little heterogeneity.

This finding partly validated other researchers’ studies. Mori and colleagues conducted a study for NACT consisting of paclitaxel and carboplatin, and they observed that patients who were non-responsive to NACT had the lowest survival rate of 50.0%, whereas the survival rate of complete responders was 100% and that of partial responders was 87.3% [37]. MacLeod and colleagues conducted a single-institutional study and found that responsiveness was a predictor of survival in univariate analysis using the Cox proportional hazards regression model, with most patients achieving an optimal response (complete response plus partial response) [38]. Selvaggi and colleagues found that in univariate Cox regression analysis, responsiveness to NACT was a significant prognostic factor of survival (*p* < 0.0001); meanwhile, the Kaplan–Meier survival curves showed that the non-responders had lower survival rates compared with complete responders and partial responders [39]. There are some limitations in the present study. First, most of the articles included in the final analysis are of retrospective nature; and the number of patients in the qualified studies varied considerably. Secondly, genetic factors may explain why some patients responded worse while others responded better [40,41]; these genetic factors had not been assessed.

## 5. Conclusions

Overall, this review shows that early non-responses to NACT are able to predict worse prognosis among LACC patients. Meanwhile, better-designed randomized control trial studies are needed to clarify the prognostic predictive value of early-stage NACT non-responses in LACC patients. This will enable clinicians to establish standards for identifying LACC patients who benefit most from NACT.

## Figures and Tables

**Table 1:** Characteristics of the included studies.

Study	Publishing Time	Location	Study Period	Population	Data Type	No. of Cases (Non-RESPONDERS)	No. of All Patients	Age at Baseline, y	Chemotherapy Cycles	FIGO Stage	Response Evaluation Guidelines	Using K-M for Data Extraction or Not	Effect Size from Cox Model or Not	Adjustment	Follow-Up Period
Robova [28]	2013	Czech Republic	1998–2009	Czech	Prospective	32	151	Mean age was 45.7 (range 20 to 70 years)	3–4	IB1-IB2	WHO Criteria	Yes	No	None	29–154 months
Liu [26]	2013	China	2002–2011	Chinese	Retrospective	40	103	Unknown	2–3	IB2/IIA2	RECIST Criteria	Yes	No	None	6–113 months
Huang [23]	2011	China	2007–2009	Chinese	Retrospective	7	52	Median (Range),46 (30 to 63)	2–3	IB2-IIA	WHO Criteria	Yes	No	None	7.1–29.3 months
Xie [30]	2016	China	2003–2008	Chinese	Retrospective	18	52	Median (Range), 43 (27 to 63)	2–3	IB2-IIB	RECIST Criteria	No	Yes	Tumor size, the expression of ALDH1	3–123 months
Yang [32]	2015	China	2007–2012	Chinese	Retrospective	33	115	Median (Range), 45 (23 to 68)	unknown	IB2-IIB	RECIST Criteria	Yes	No	None	6–75 months
Park [27]	2011	Korean	1997–2007	Chinese	Prospective	15	43	Median (Range), 50 (30 to 78)	Mostly 3	IIB	RECIST Criteria	No	Yes	Node, the expression of ERCC1	6–139 months
Li [24]	2012	China	2000–2011	Chinese	Retrospective	43	154	Mean ± SD, 41.6 ± 7.92	2–3	IB2/IIA2	RECIST Criteria	Yes	No	None	6–142 months
Shoji [29]	2013	Japan	2002–2011	Japanese	Pilot study	5	23	Median (Range), 50 (32 to 63)	1–3	IB2-IIB	RECIST Criteria	No	Yes	None	9–90 months
Li (1) [25]	2016	China	1999–2008	Chinese	Retrospective	189	826	Median (Range), 44 (39 to 50)	Mostly 1–2	IB2-IIB	WHO Criteria	No	Yes	Age, Stage, Tumor size, Grade, Cell type, LVSI, Parametrial infiltration, Vaginal surgical margin, Lymph node metastasis	0–115 months
Li (2) [25]	2016	China	2003–2013	Chinese	Prospective	115	485	Median (Range), 45 (40 to 49)	Mostly 1–2	IB2-IIB	WHO Criteria	No	Yes	Age, Stage, Tumor size, Grade, Cell type, LVSI, Parametrial infiltration, Vaginal surgical margin, Lymph node metastasis	0–100 months
Xiong [31]	2011	China	2004–2009	Chinese	Retrospective	21	60	Median (Range), 49 (28–61)	2	IB2-IIB	WHO criteria	No	No	None	7–66 months

Abbr.: ALDH1, Aldehyde dehydrogenas 1; ERCC1, Excision repair cross-complementation gene group 1; LVSI, Lymph vascular space invasion.

## Data Availability

No new data were created or analyzed in this study. Data sharing is not applicable to this article.

## Supplementary Materials

The following supporting information can be downloaded at: https://www.mdpi.com/article/10.3390/biomedicines13082016/s1. Figure S1. Publication bias detection including Funnel plot, Egger's regression, Begg's test, and Trim-and-Fill analysis. Figure S2. Sensitivity analysis for disease-free survival HR robustness. Figure S3. Multi-method evaluation of publication bias in random-effects pooled analysis of hazard ratios. Figure S4. Sensitivity analysis for HR robustness in DFS outcomes. Figure S5. Publication bias detection including Funnel plot, Egger's regression, Begg's test, and Trim-and-Fill analysis of the WHO studies using fixed-effect model. Figure S6. Sensitivity analysis for disease-free survival HR robustness for WHO studies using fixed-effect model. Figure S7. Publication bias detection including Funnel plot, Egger's regression, Begg's test, and Trim-and-Fill analysis of the WHO studies using random effect model. Figure S8. Sensitivity analysis for disease-free survival HR robustness for WHO studies using random effect model. Figure S9. Publication bias detection including Funnel plot, Egger's regression, Begg's test, and Trim-and-Fill analysis of the RECIST studies using fixed effect model. Figure S10. Sensitivity analysis for disease-free survival HR robustness for RECIST studies using fixed-effect model. Figure S11. Publication bias detection including Funnel plot, Egger's regression, Begg's test, and Trim-and-Fill analysis of the RECIST studies using random effect model. Figure S12. Sensitivity analysis for disease-free survival HR robustness for RECIST studies using random effect model. Figure S13. Publication bias detection including Funnel plot, Egger's regression, Begg's test, and Trim-and-Fill analysis of the Asian studies using fixed effect model. Figure S14. Sensitivity analysis for disease-free survival HR robustness for RECIST studies using fixed effect model. Figure S15. Publication bias detection including Funnel plot, Egger's regression, Begg's test, and Trim-and-Fill analysis of the Asian studies using random effect model. Figure S16. Sensitivity analysis for disease-free survival HR robustness for RECIST studies using random effect model. Figure S17. Publication bias detection including Funnel plot, Egger's regression, Begg's test, and Trim-and-Fill analysis of the Asian WHO studies using fixed effect model. Figure S18. Sensitivity analysis for disease-free survival HR robustness for Asian WHO studies using fixed effect model. Figure S19. Publication bias detection including Funnel plot, Egger's regression, Begg's test, and Trim-and-Fill analysis of the Asian WHO studies using random effect model. Figure S20. Sensitivity analysis for disease-free survival HR robustness for Asian WHO studies using random effect model.

## Abbreviations

The following abbreviations are used in this manuscript:

LACC	Locally advanced cervical cancer
NACT	Neoadjuvant chemotherapy
DFS	Disease-free survival
RFS	Recurrence-free survival
HR	Hazard ratio
CI	Confidence interval
RECIST	Response evaluation criteria in solid tumors
ALDH1	Aldehyde dehydrogenas 1
ERCC1	Excision repair cross-complementation gene group 1
LVSI	Lymph-vascular space invasion
