# Supplementary material for: Early Non-Response to Neoadjuvant Chemotherapy Will Increase the Recurrence of Cervical Cancer: A Systematic Review
Source: Biomedicines. 2025 Aug 19;13(8):2016. doi: 10.3390/biomedicines13082016 (PMC12383554; doi:10.3390/biomedicines13082016)
Supplement: Supplementary file 1 [file biomedicines-13-02016-s001.zip › biomedicines-3677554-supplementary.pdf]

Supplementary Materials:

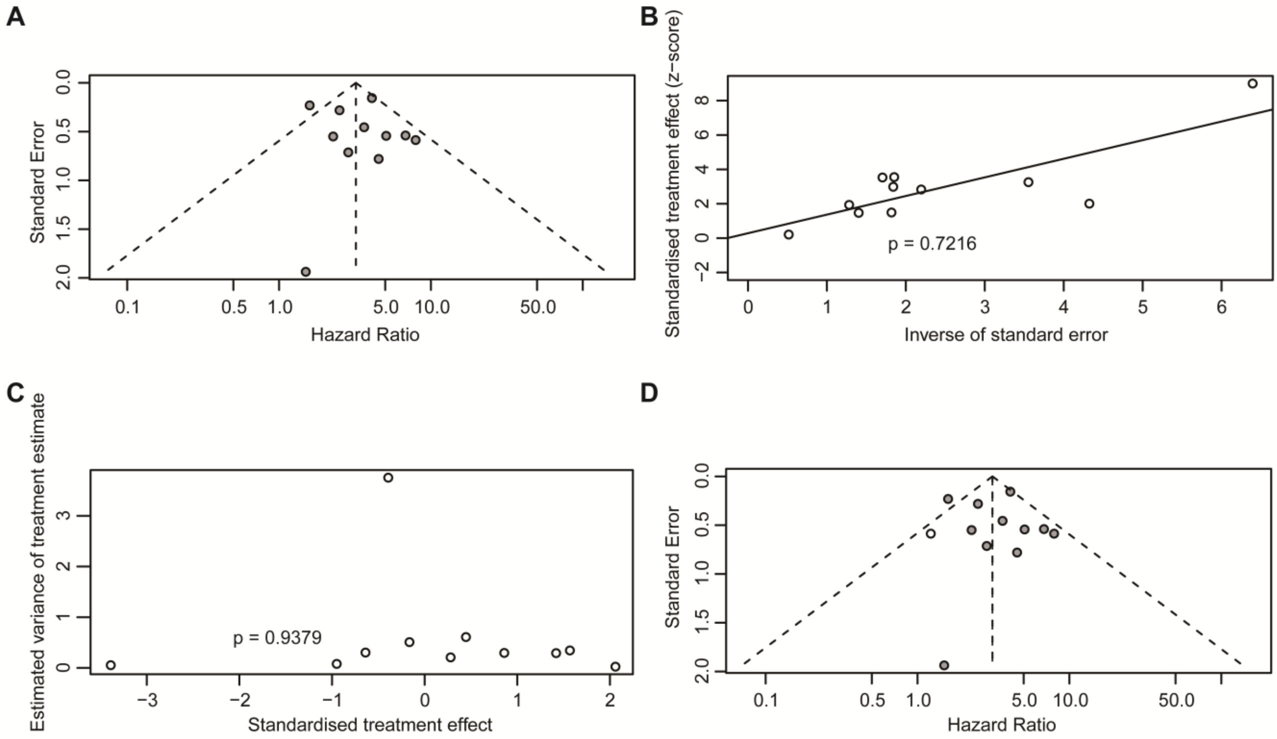

Figure S1. Title: Publication bias detection including Funnel plot, Egger's regression, Begg's test, and Trim-and-Fill analysis. Legend: This methodological analysis employs four complementary techniques to assess publication bias in meta-analyses of hazard ratios (HRs) using fixed-effect models: (A) Funnel plot; (B) Egger's regression test; (C) Begg's rank correlation test; (D) Trim-and-Fill method.

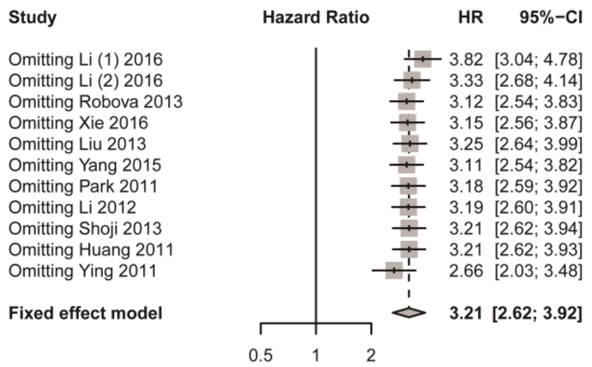

Figure S2. Title: Sensitivity analysis for disease-free survival HR robustness. Legend: This methodological assessment evaluates the stability of DFS hazard ratios (HRs) under fixed-effect modeling through systematic sensitivity testing.

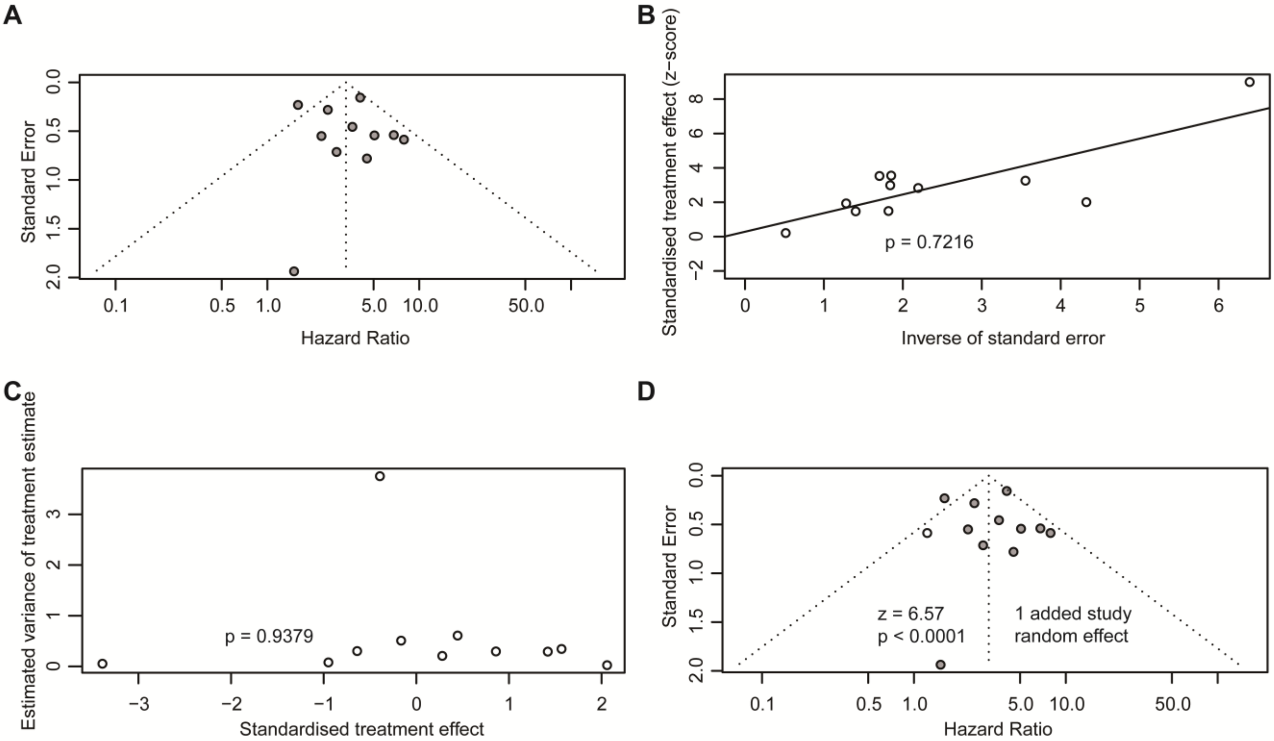

Figure S3. Title: Multi-method evaluation of publication bias in random-effects pooled analysis of hazard ratios. Legend: This methodological evaluation integrates four established techniques to assess publication bias in pooled analyses of hazard ratios (HRs) utilizing random-effects models: (A) Funnel plot analysis; (B) Egger's regression test; (C) Begg's rank correlation test; (D) Trim-and-Fill procedure.

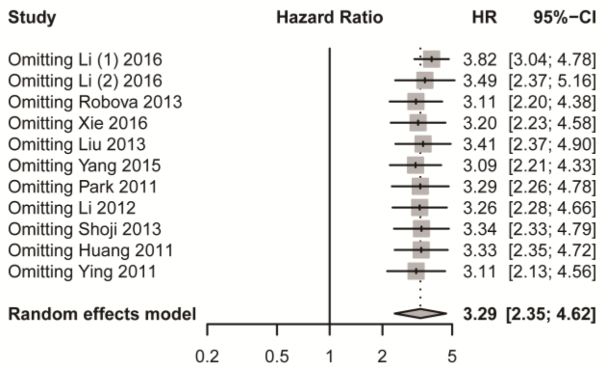

Figure S4. Title: Sensitivity analysis for HR robustness in DFS outcomes. Legend: This methodological framework evaluates the stability of HR estimates for DFS through systematic sensitivity testing using fixed-effect modeling.

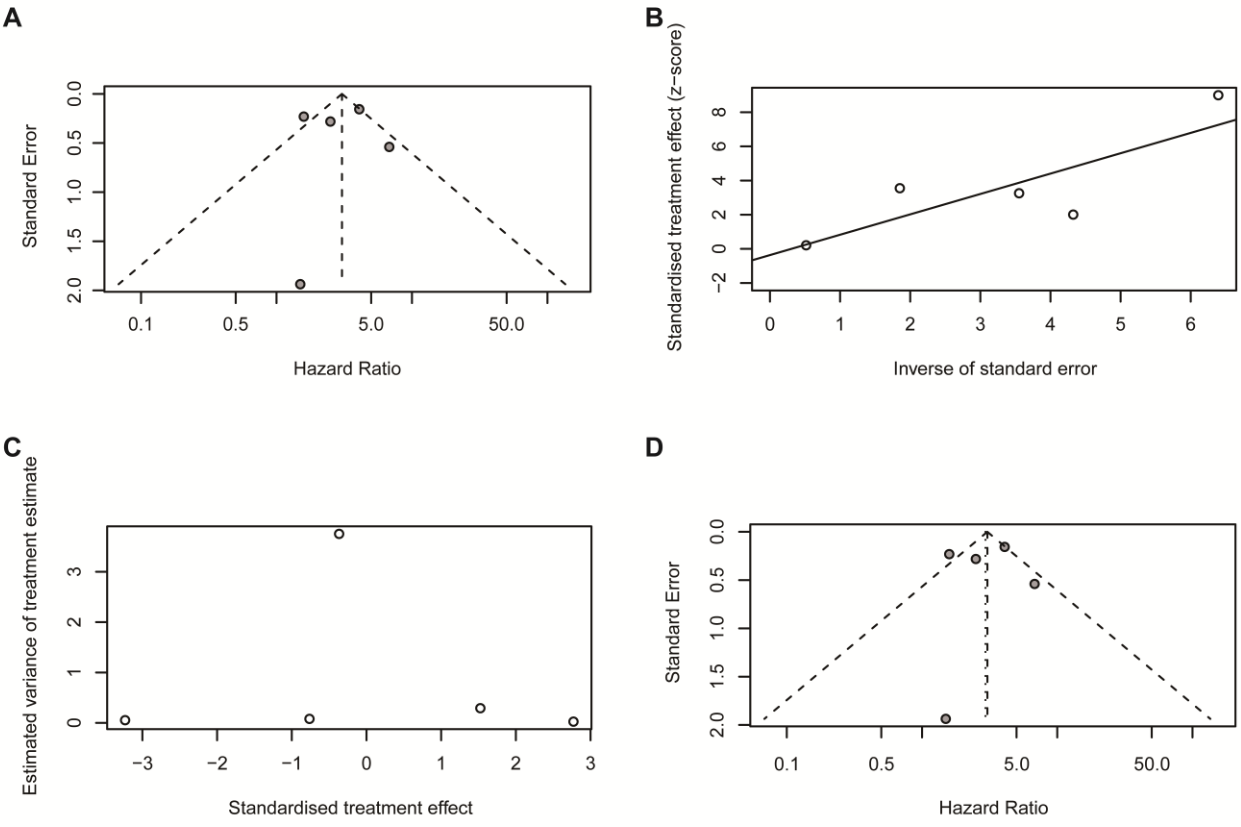

Figure S5. Title: Publication bias detection including Funnel plot, Egger's regression, Begg's test, and Trim-and-Fill analysis of the WHO studies using fixed-effect model. Legend: This methodological analysis employs four complementary techniques to assess publication bias in meta-analyses of hazard ratios (HRs) using fixed-effect models: (A) Funnel plot; (B) Egger's regression test; (C) Begg's rank correlation test; (D) Trim-and-Fill method.

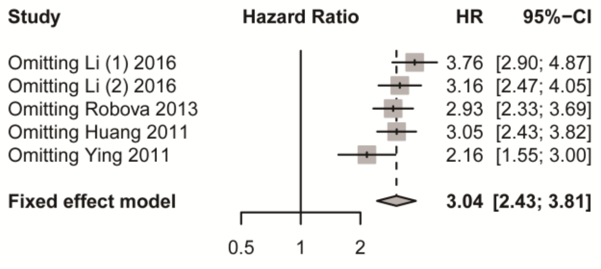

Figure S6. Title: Sensitivity analysis for disease-free survival HR robustness for WHO studies using fixed-effect model. Legend: This methodological assessment evaluates the stability of DFS hazard ratios (HRs) under fixed-effect modeling through systematic sensitivity testing.

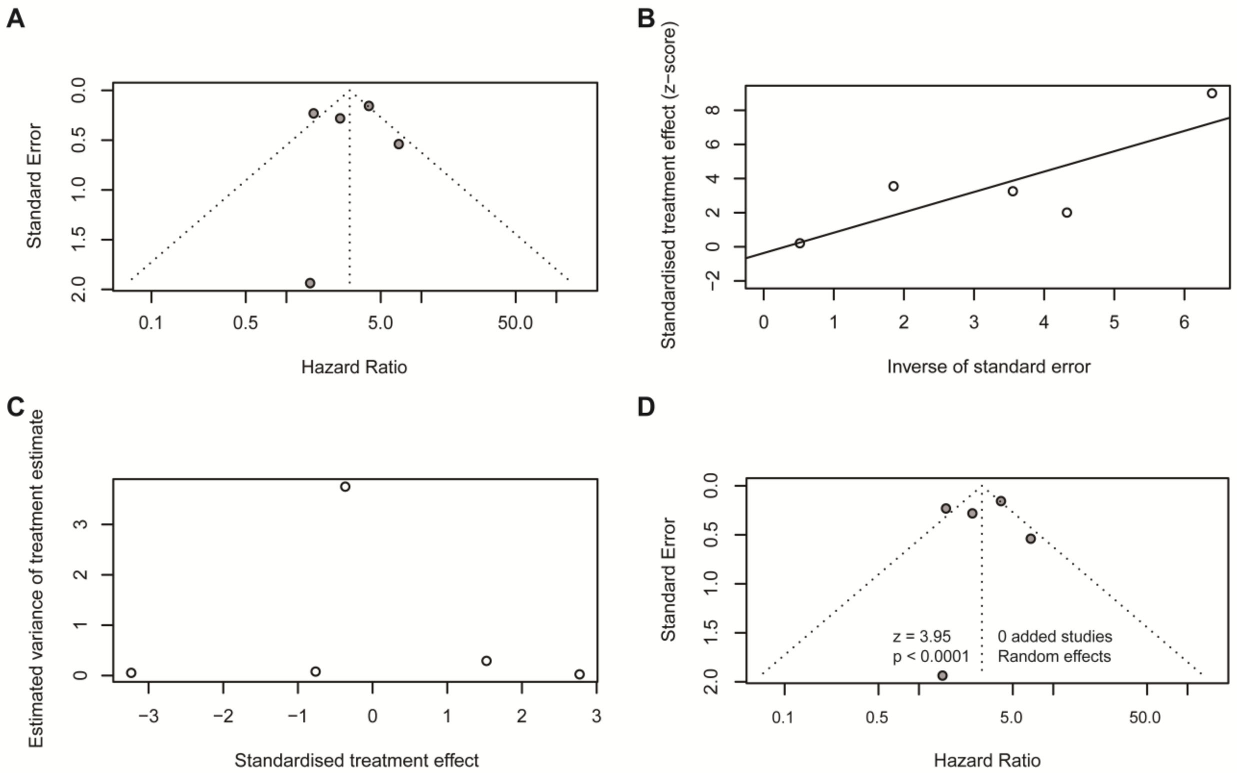

Figure S7. Title: Publication bias detection including Funnel plot, Egger's regression, Begg's test, and Trim-and-Fill analysis of the WHO studies using random effect model. Legend: This methodological analysis employs four complementary techniques to assess publication bias in meta-analyses of hazard ratios (HRs) using fixed-effect models: (A) Funnel plot; (B) Egger's regression test; (C) Begg's rank correlation test; (D) Trim-and-Fill method.

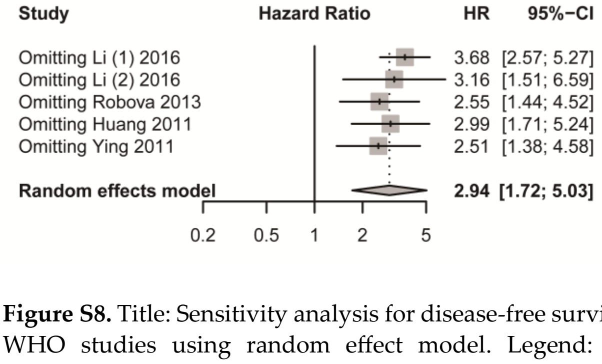

Figure S8. Title: Sensitivity analysis for disease-free survival HR robustness for WHO studies using random effect model. Legend: This methodological assessment evaluates the stability of DFS hazard ratios (HRs) under fixed-effect modeling through systematic sensitivity testing.

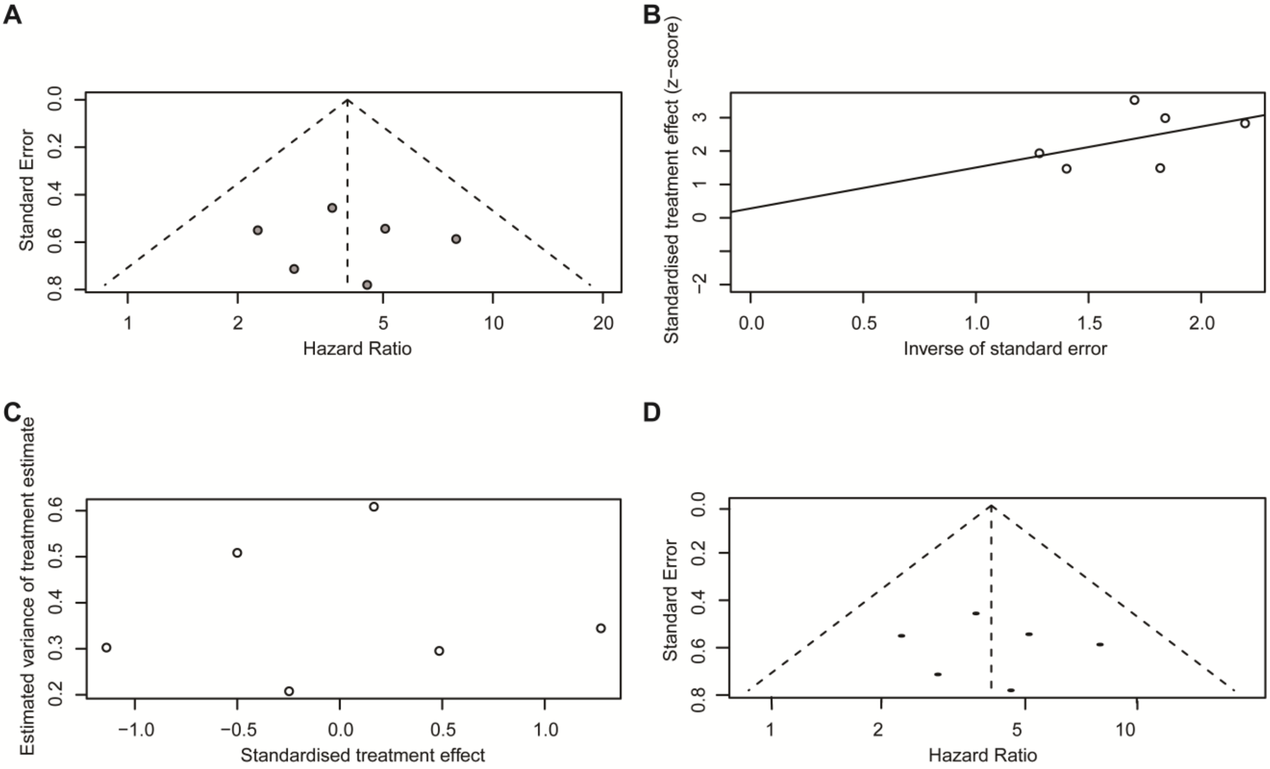

Figure S9. Title: Publication bias detection including Funnel plot, Egger's regression, Begg's test, and Trim-and-Fill analysis of the RECIST studies using fixed effect model. Legend: This methodological analysis employs four complementary techniques to assess publication bias in meta-analyses of hazard ratios (HRs) using fixed-effect models: (A) Funnel plot; (B) Egger's regression test; (C) Begg's rank correlation test; (D) Trim-and-Fill method.

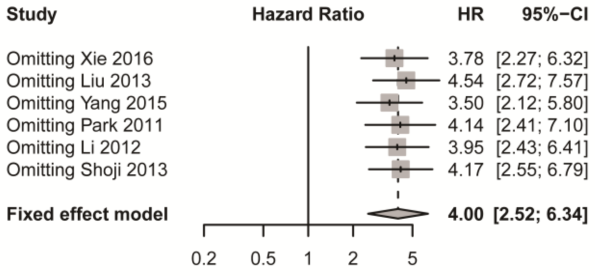

Figure S10. Title: Sensitivity analysis for disease-free survival HR robustness for RECIST studies using fixed-effect model. Legend: This methodological assessment evaluates the stability of DFS hazard ratios (HRs) under fixed-effect modeling through systematic sensitivity testing.

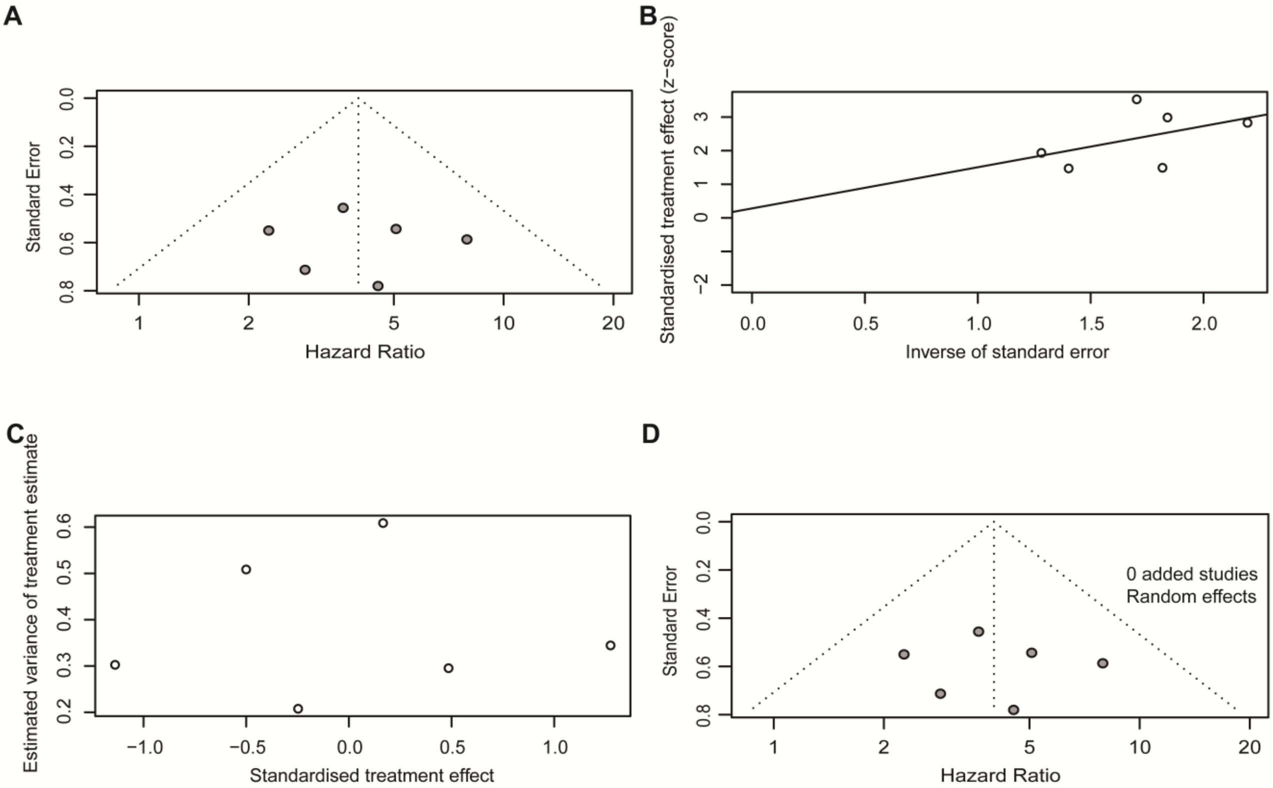

Figure S11. Title: Publication bias detection including Funnel plot, Egger's regression, Begg's test, and Trim-and-Fill analysis of the RECIST studies using random effect model. Legend: This methodological analysis employs four complementary techniques to assess publication bias in meta-analyses of hazard ratios (HRs) using fixed-effect models: (A) Funnel plot; (B) Egger's regression test; (C) Begg's rank correlation test; (D) Trim-and-Fill method.

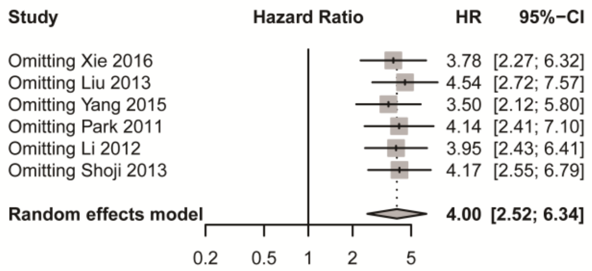

Figure S12. Title: Sensitivity analysis for disease-free survival HR robustness for RECIST studies using random effect model. Legend: This methodological assessment evaluates the stability of DFS hazard ratios (HRs) under fixed-effect modeling through systematic sensitivity testing.

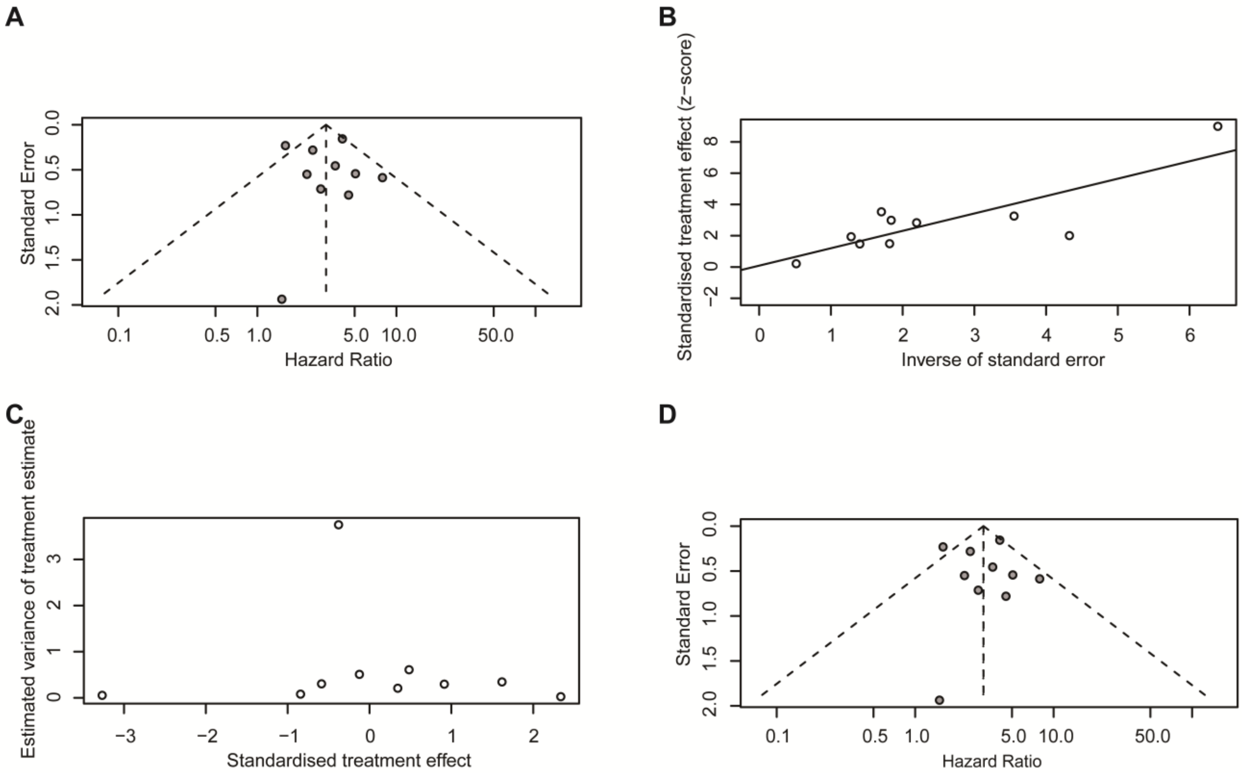

Figure S13. Title: Publication bias detection including Funnel plot, Egger's regression, Begg's test, and Trim-and-Fill analysis of the Asian studies using fixed effect model. Legend: This methodological analysis employs four complementary techniques to assess publication bias in meta-analyses of hazard ratios (HRs) using fixed-effect models: (A) Funnel plot; (B) Egger's regression test; (C) Begg's rank correlation test; (D) Trim-and-Fill method.

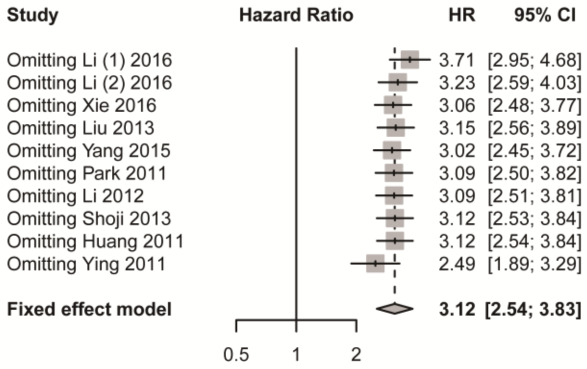

Figure S14. Title: Sensitivity analysis for disease-free survival HR robustness for RECIST studies using fixed effect model. Legend: This methodological assessment evaluates the stability of DFS hazard ratios (HRs) under fixed-effect modeling through systematic sensitivity testing.

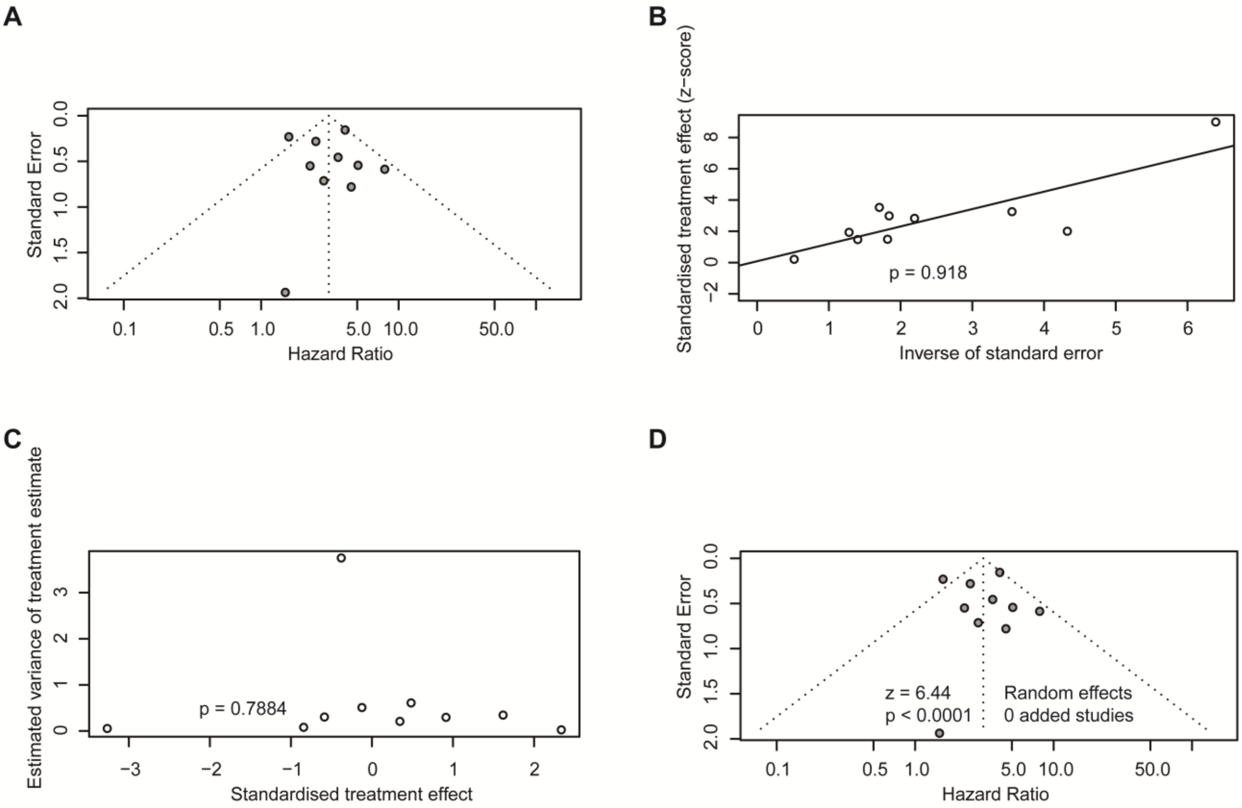

Figure S15. Title: Publication bias detection including Funnel plot, Egger's regression, Begg's test, and Trim-and-Fill analysis of the Asian studies using random effect model. Legend: This methodological analysis employs four complementary techniques to assess publication bias in meta-analyses of hazard ratios (HRs) using fixed-effect models: (A) Funnel plot; (B) Egger's regression test; (C) Begg's rank correlation test; (D) Trim-and-Fill method.

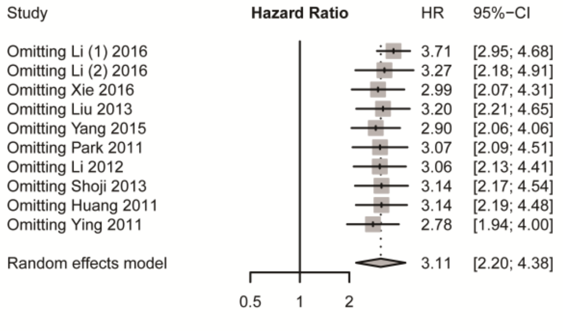

Figure S16. Title: Sensitivity analysis for disease-free survival HR robustness for RECIST studies using random effect model. Legend: This methodological assessment evaluates the stability of DFS hazard ratios (HRs) under fixed-effect modeling through systematic sensitivity testing.

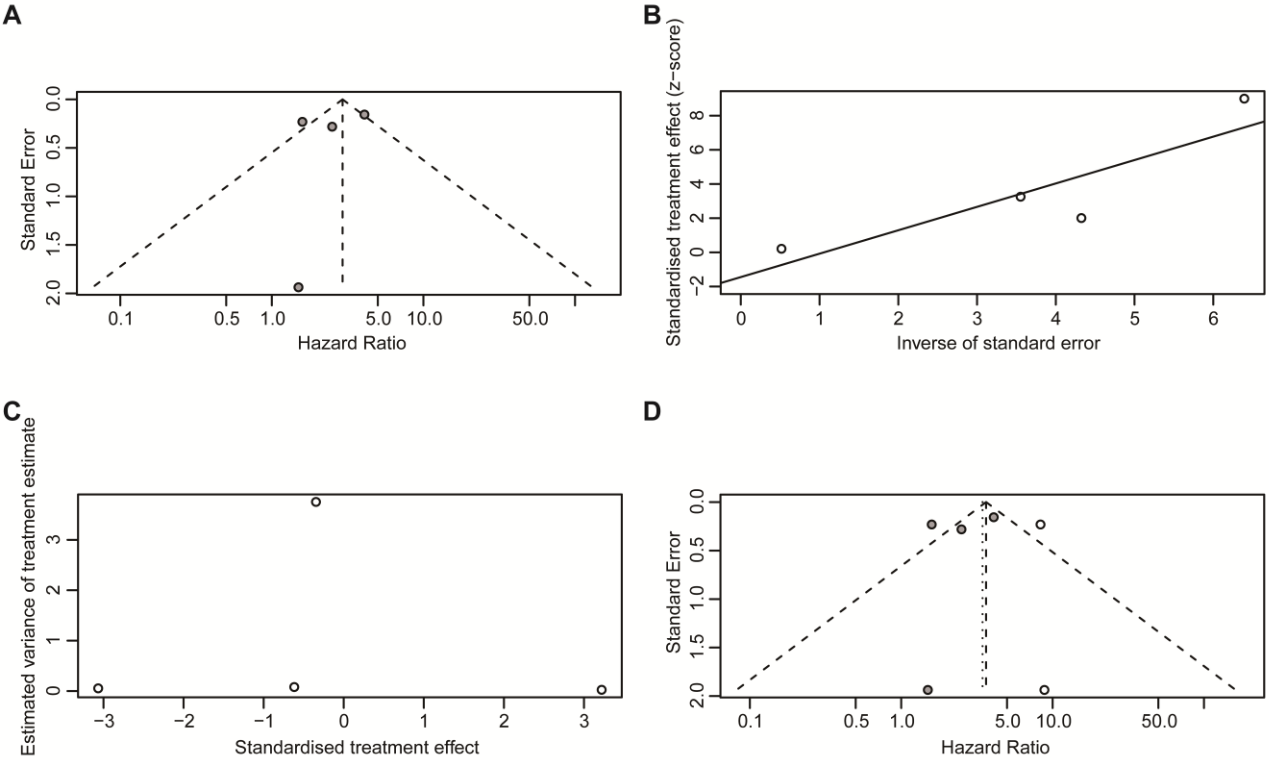

Figure S17. Title: Publication bias detection including Funnel plot, Egger's regression, Begg's test, and Trim-and-Fill analysis of the Asian WHO studies using fixed effect model. Legend: This methodological analysis employs four complementary techniques to assess publication bias in meta-analyses of hazard ratios (HRs) using fixed-effect models: (A) Funnel plot; (B) Egger's regression test; (C) Begg's rank correlation test; (D) Trim-and-Fill method.

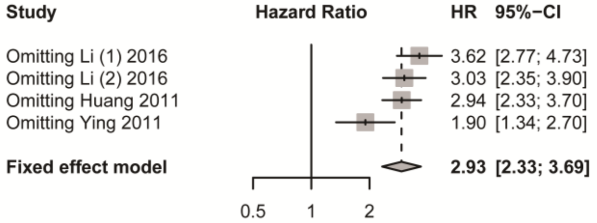

Figure S18. Title: Sensitivity analysis for disease-free survival HR robustness for Asian WHO studies using fixed effect model. Legend: This methodological assessment evaluates the stability of DFS hazard ratios (HRs) under fixed-effect modeling through systematic sensitivity testing.

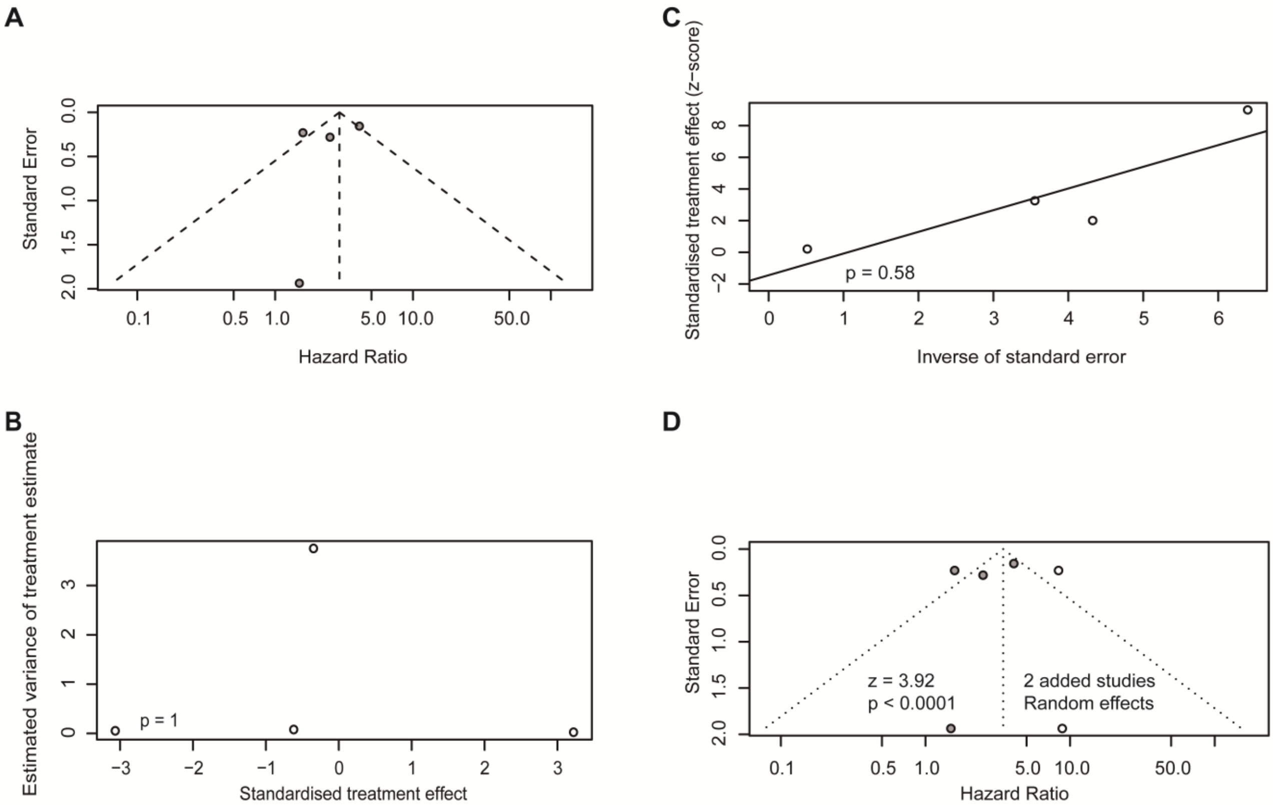

Figure S19. Title: Publication bias detection including Funnel plot, Egger's regression, Begg's test, and Trim-and-Fill analysis of the Asian WHO studies using random effect model. Legend: This methodological analysis employs four complementary techniques to assess publication bias in meta-analyses of hazard ratios (HRs) using fixed-effect models: (A) Funnel plot; (B) Egger's regression test; (C) Begg's rank correlation test; (D) Trim-and-Fill method.

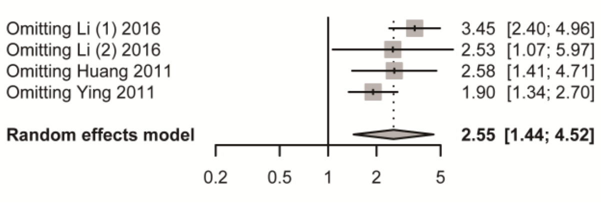

Figure S20. Title: Sensitivity analysis for disease-free survival HR robustness for Asian WHO studies using random effect model. Legend: This methodological assessment evaluates the stability of DFS hazard ratios (HRs) under fixed-effect modeling through systematic sensitivity testing.
